# CANNABIS USE BY CAREGIVERS OF PERSONS WITH DEMENTIA

**DOI:** 10.1093/geroni/igz038.739

**Published:** 2019-11-08

**Authors:** Freddi Segal-Gidan

**Affiliations:** University of Southern California, Los Angeles, California, United States

## Abstract

Thirty three states have legalized marijuana for medical or recreational use. This increased access to cannabis products has heightened interest in its use among family caregivers. Clinicians anecdotally report frequent questions from families about the use of these products, but lack sound information to provide an informed response. Some studies suggest cannabis could help to manage behavioral symptoms of dementia,. No studies to date have explored marijuana or cannabis product use by caregivers for themselves or for the person with dementia. Caregivers of persons with diagnosed dementia were invited to take part in a study that included completion of a survey and participation in a focus group that explored self care practices, including the use of marijuana and cannabidiol (CBD) products for themselves and the person with dementia. Results of the survey and trends from the focus groups will be presented, with a comparison between English and Spanish speaking individuals.

